# In search of attachment: a qualitative study of chronically ill women transitioning between family physicians in rural Ontario, Canada

**DOI:** 10.1186/1471-2296-13-125

**Published:** 2012-12-23

**Authors:** Ellen Randall, Valorie A Crooks, Laurie J Goldsmith

**Affiliations:** 1 Faculty of Health Sciences, Simon Fraser University, 8888 University Drive, Burnaby, BC, V5A 1S6, Canada; 2 Department of Geography, Simon Fraser University, 8888 University Drive, Burnaby, BC, V5A 1S6, Canada

**Keywords:** Family medicine, Canada, Unattached patients, Continuity, Attachment, Rural

## Abstract

**Background:**

Most Canadians receive basic health services from a family physician and these physicians are particularly critical in the management of chronic disease. Canada, however, has an endemic shortage of family physicians. Physician shortages and turnover are particularly acute in rural regions, leaving their residents at risk of needing to transition between family physicians. The knowledge base about how patients manage transitioning in a climate of scarcity remains nascent. The purpose of this study is to explore the experience of transitioning for chronically ill, rurally situated Canadian women to provide insight into if and how the system supports transitioning patients and to identify opportunities for enhancing that support.

**Methods:**

Chronically ill women managing rheumatic diseases residing in two rural counties in the province of Ontario were recruited to participate in face-to-face, semi-structured interviews. Interview transcripts were analysed thematically to identify emergent themes associated with the transitioning experience.

**Results:**

Seventeen women participated in this study. Ten had experienced transitioning and four with long-standing family physicians anticipated doing so soon. The remaining three expressed concerns about transitioning. Thematic analysis revealed the presence of a transitioning trajectory with three phases. The detachment phase focused on activities related to the termination of a physician-patient relationship, including haphazard notification tactics and the absence of referrals to replacement physicians. For those unable to immediately find a new doctor, there was a phase of unattachment during which patients had to improvise ways to receive care from alternative providers or walk-in clinics. The final phase, attachment, was characterized by acceptance into the practice of a new family physician.

**Conclusions:**

Participants often found transitioning challenging, largely due to perceived gaps in support from the health care system. Barriers to a smooth transition included inadequate notification procedures, lack of formal assistance finding new physicians, and unsatisfactory experiences seeking care during unattachment. The participants’ accounts reveal opportunities for a stronger system presence during transition and a need for further research into alternative models of primary care delivery.

## Background

For most Canadians the usual means of engaging with the health care system is through an on-going relationship with a regular family physician, also known as ‘attachment’ [1]. This practice is reinforced by the system’s design which designates the family physician as chief steward of care over the life course and gatekeeper to specialist care [2]. Canada, however, like many countries, is grappling with a shortage of family physicians for reasons varying from a growing lack of interest in family practice to changes in practice patterns [3-6]. At any given time over the last decade over four million Canadians were without a regular doctor [7-9], due in large part to an estimated deficit of roughly 3200 family physicians [10]. This deficit is particularly acute in rural regions where on-going challenges with recruitment and turnover limit physician supply [11-14]. While 21% of Canadians live in rural areas, only 16% of family physicians practice in rural communities [12]. Rural rates of physician turnover, often linked to isolation and high workload, can be as much as 40-100% higher than in urban areas [13-15]. As a consequence, rural Canadian patients are at particular risk of needing to transition – to detach from one family physician and then source and be accepted into the practice of another. With an insufficient physician supply, this can be a challenging proposition.

Given the centrality of the family physician to the delivery of primary care in Canada, patients seeking on-going and preventive care, as well as specialist referrals, are best served through attachment to a family physician [2]. Patients who are not attached have been found to be at risk for reduced receipt of preventive care and screening [16,17]. The traditional patient-family physician dyad, on the other hand, is seen as key to ensuring continuity of care [18], long considered an indicator of good health care [19,20]. There is a solid body of evidence pointing to the benefits associated with having a usual family physician, including more timely system entry [21,22], more preventive care [21,23,24], better rates of screening [25], and greater patient satisfaction [26] when compared to patients who lack a regular family physician.

In this study, rural communities are understood as places with small populations, limited material and financial resources, and a heightened vulnerability to health service and health human resource shortages as a consequence of their distance from urban centres [12,27-29]. Given the small numbers of rurally-based specialists, there may be an added benefit for rural patients who have a regular family physician. With specialists concentrated in urban areas, some rural family physicians expand their scope of practice to encompass at least a portion of the services patients might normally receive from a specialist, including aspects of chronic disease management [12]. Rural communities tend to have a high proportion of seniors [30], a group at risk for chronic disease [31]. And in Canada, as around the world, the number of patients managing chronic illnesses is on the rise [32-34]. Roughly a third of Canadians will develop at least one chronic disease, with prevalence rising to 75% among those over 65 [31]. It is reasonable, therefore, to expect a high demand for chronic disease care in rural Canada.

Chronically ill patients tend to have high needs for primary and specialist care [35]. In the Canadian system, it is the family physician who is best positioned to coordinate an integrated care plan across the full spectrum of health services – the foundation of optimal chronic disease management [36,37]. Health service utilization patterns confirm this reliance, with chronically ill patients accounting for 51% of family physician consults [31]. Rural communities, therefore, are often in the unique position of contending with the simultaneous pressures of an aging population, rising rates of chronic illness, and endemic family physician shortages – all of which combine to put chronically ill, rural patients at risk for inadequate receipt of needed care.

For Canadians who detach from a family physician there is no guarantee of being referred to, or easily finding, a new regular physician. In a climate of family physician scarcity, these patients may enter a process of searching for attachment that includes a protracted period without a usual doctor [38,39]. In rural communities this separation is often triggered by turnover or retirement which can leave an entire practice of patients unattached and looking for new physicians at the same time [38,40]. Many Canadian provinces issue guidelines regarding practice closure but their recommendations about assisting patients in finding new physicians vary [41-44], and it is unclear if and how these guidelines are being implemented by physicians. While there is evidence that physician turnover is associated with reduced patient satisfaction [45,46], there is a relative dearth of research exploring this phenomenon and its effect on patients [45]. And little is known about the experience of being unattached for a prolonged period of time which, in a climate of physician shortage, may happen following the end of a relationship with a family physician [47,48]. There is scant understanding, for example, of how patients who are unattached manage care [47,48], although it is believed they rely on episodic care through walk-in clinics and emergency rooms [7,49]. For most Canadians, however, managing care this way is not optimal [7]. The ultimate goal for most unattached patients is attachment to a new doctor and a majority of patients express a preference for an on-going relationship with a regular physician [50-52].

Empirical understanding of how patients detach from family physicians and move forward to a new attachment remains nascent. In this article we identify and examine common elements in this transition for chronically ill women managing rheumatic diseases in rural Ontario, Canada. These women live in communities where endemically low supplies of family physicians, exacerbated by high rates of turnover [11-15], can leave patients at risk of losing and having to source family doctors [13-15]. As Canadian health policymakers work to facilitate attachment and system use through a variety of primary care reform initiatives [53-56], there is a need to inform system performance evaluation and policy development with an understanding of the patient experience [57-60]. With a large population of unattached Canadian patients in the midst of a family physician shortage, we contend that understanding the experiences of these individuals will provide policymakers and clinicians with needed insight into if and how the system supports rural residents faced with transitioning. This insight will provide an opportunity to explore short- and long-term possibilities for mitigating the challenges these patients encounter along the way.

## Methods

The goal of this qualitative study was to explore the experiences of rurally situated women managing rheumatic disease (e.g., osteoarthritis, lupus, rheumatoid arthritis, fibromyalgia syndrome, scleroderma, gout, bursitis) who had experienced, or anticipated, family physician turnover and needing to transition to a new regular doctor. Patients with rheumatic diseases were selected because of the prevalence of these conditions [31] and because management of this group of diseases can largely be undertaken at the primary care level [61,62], heightening the importance of attachment to a family doctor. Female patients were targeted because they experience most rheumatic diseases at higher rates than men [31,63] and they tend to place higher value on the continuity of care that can be an outcome of attachment, than male patients [64]. The participants resided in Grey and Bruce Counties in Ontario, Canada. These counties were chosen because, like many rural areas in Canada, they have experienced a family physician shortage compounded by a high rate of physician turnover or retirement [65,66], elevating the likelihood that local patients would, at some point, experience the loss of a family physician and need to transition to a replacement.

Given the exploratory nature of this study, and the goal of capturing the patient perspective on transitioning, a qualitative approach was employed. Data were gathered by means of face-to-face, semi-structured interviews which allowed the experience of transitioning to emerge in a patient’s own words and reflect her individual sense of what was significant. As a patient’s experience with her health and health practitioner may touch upon sensitive issues, it was thought that the use of one-on-one conversation would enable the establishment of rapport and encourage the sharing of personal reflections [67]. Approval for the study was granted by the Office of Research Services at York University and by the Office of Research Ethics at Simon Fraser University.

### Recruitment

The study used a purposive sampling frame, targeting women over the age of 18 living in Grey and Bruce Counties who self-identified as managing at least one rheumatic disease and who had experienced a turnover in their family physicians, or were lacking a regular doctor altogether. Using this frame, participants were recruited opportunistically with the assistance of The Arthritis Society of Ontario, which mailed letters to its members in the pertinent counties containing information about the study, along with dates for the data collection period and contact details for the interviewer. Those interested in participating were asked to contact the interviewer for further information and to schedule a time and location for a face-to-face meeting. Eighteen women scheduled interviews within the data collection period.

### Data collection

Data collection took place over a three-week period, and all women who had scheduled an interview participated. Semi-structured interviews were selected as the method for data collection. This interview format enables the collection of both historical and current information [68] and allows for the development of a set of framing questions that remain sufficiently open-ended to allow respondents the freedom and flexibility to express their meanings in their own way [69]. This flexibility also enables the interviewer to develop new questions and frame follow-up questions using respondents’ terms of reference [70]. The interviews were conducted by the second author and took place in a location of the participant’s choosing. At the time of the interview, participants were offered the opportunity to select a pseudonym to be used as the unique identifier for their transcripts; all participants elected to do so.

Written informed consent for participation in the study was obtained at the outset of the interview. Interviews lasted roughly an hour. Two semi-structured interview guides were developed – one for participants who had experienced family physician turnover, and one for those who had not but expressed concern about needing to do so in the near future. The basic framework for these guides was identical, sharing four principal categories of questions: (1) health status and history (e.g. health information); (2) health care practitioner history (e.g. frequency of visits to family physician); (3) implications of transitioning (e.g. implications of detachment/discontinuity); and (4) demographics (e.g. employment status). These common categories ensured that all interviews covered the same basic ground, while the open-ended questions allowed both interviewer and respondent the flexibility to explore related content areas [67,71]. The interview guide for those who had experienced physician turnover contained additional questions regarding participants’ experiences transitioning and, if relevant, how they managed to receive primary care services during the period they were without a regular doctor.

### Data analysis

All interviews were recorded and transcribed verbatim. After an initial transcript review it became apparent that one participant who had indicated that she met the inclusion criteria, had not experienced or anticipated family physician turnover. This participant was excluded from the analysis, bringing the study sample size to 17 participants. De-identified demographic data were entered into a spreadsheet to create a descriptive profile of each participant. Transcripts and profiles were identified by participants’ chosen pseudonyms, allowing them to be linked. After a preliminary review of the transcripts, a thematic analysis was undertaken. Given this was an exploratory study seeking to identify common elements of a shared experience, thematic analysis enabled a data-driven, inductive approach that allowed for the identification of first macro and then more granular themes associated with the process of transitioning from one physician to another [72].

The first step in the analytic process involved transcript reviews by the first and second authors. Specifically, a subset of the transcripts was systematically reviewed to identify and describe emergent themes. Following independent review, the investigators came together to discuss dominant themes and their interpretation. Many themes were “indicated by the data” [73], wherein key concepts were embedded in participants’ accounts of transitioning or their concerns about needing to transition in the future. An important emergent concept was that of the ‘transitioning trajectory’, which involves three temporally-driven themes, each reflecting a different phase in the trajectory.

After consensus was reached between the first and second authors about the scope of the analytic focus, an interpretive matrix was developed that outlined the structure of the analysis and identified distinct experiences that characterized the themes associated with transitioning. This matrix was reviewed and confirmed by all three authors. A coding schema was then developed by the first author with input from the second author, labelling and defining the basic thematic and sub-thematic categories, and giving guidance about inclusion and exclusion in order to prevent drift during coding [68,72]. Coding was undertaken in a word processing program and data central to each code were extracted and combined in separate documents.

Following coding, the first author identified data extracts that best characterized the set of distinct experiences that made up each of the three temporally-driven themes and further refined the interpretive matrix. Relationships within and across themes were established through a review of the extracts and the populated matrix. To enhance the rigour of the analytic process, the second and third authors reviewed summative forms of the analysis, and the extracts were reviewed by the second author to confirm interpretations. The findings were also compared to the existing academic literature and print news media. Patient accounts in the mass media affirmed the experiences of the study participants [8,74], while the academic literature was limited and tended not to deal with the full transitioning process.

The quotes shared in the results section are drawn from the extracts included in the fully populated interpretive matrix. Pseudonyms selected by participants are used when quotes are provided in order to acknowledge and personalize the women’s direct contributions.

## Results

Seventeen women participated in the study. They varied in age from 39 to 87 years and two-thirds were between 44 and 62 years old. All participants self-identified as being chronically ill with at least one type of rheumatic disease and the majority described having more than one health issue. Osteoarthritis, rheumatoid arthritis and fibromyalgia syndrome were the most commonly cited rheumatic diseases. Eight women were not working and received disability benefits; the remainder were employed or in retirement. Ten participants reported having transitioned from at least one family physician within the preceding decade; seven of those ten had transitioned two or more times. In most instances, transitions had been, or were going to be, initiated by the physician due to retirement, relocation, a switch in practice focus, or physician illness. The seven women who had not recently transitioned had long-term relationships (15–32 years) with their current family physicians. These women fell into two categories: those who were expecting to transition and those who sought means to avoid transitioning. Four of these seven women actively anticipated transitioning in the near future due to the imminent retirement of their doctors. The other three elected to adopt means to sustain a long-term relationship and avoid detachment: two saw practitioners in neighbouring communities as a way around having to find a local doctor, and one remained with a local physician she was not satisfied with for fear of not finding another.

**Figure 1:**
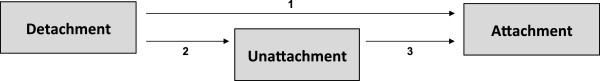
**The Transitioning Trajectory.** The transitioning trajectory consists of two or three phases. Patients who detach and immediately source an available family physician go directly from detachment to attachment (**1**). Patients who spend a period without a physician while searching for a one pass through unattachment en route to attachment (**2**). For this group attachment can only be achieved if a new regular physician is sourced (**3**).

In the remainder of this section we examine the trajectory that dominated participants’ discussions of transitioning between family physicians. Although not asked explicitly to comment on phases associated with transitioning, participants routinely and consistently described elements of three distinct, temporally-driven phases. The concept of a multi-phase *transitioning trajectory* emerged from these accounts of their experiences (see Figure 1). Participants who could immediately identify an available family physician experienced two transitioning phases, while those who were unable to immediately find a new doctor experienced three. Descriptions of the phase of *detachment* encompassed activities related to the termination of a physician-patient relationship by the physician. For the participants who struggled to find a new family physician, there was a phase of *unattachment* during which patients were without a regular family physician and had to find alternative means of receiving primary care. This phase was also often characterized by unsuccessful attempts to find a new doctor. The final phase was *attachment*, during which patients successfully sourced a new physician and were accepted into her or his practice.

### Detachment phase

Several participants acknowledged the connection between physician scarcity and their place of residence, with one woman noting that there “weren’t enough doctors around” (Barbara). These women were aware that detachment from a family physician in a rural area with a physician shortage and high physician turnover might lead to difficulties finding a replacement, and therefore was something to be avoided. Three participants described conscious choices to avoid detachment. Referencing the local doctor shortage one woman explained that she chose not to detach from her family physician, despite being dissatisfied with her care experience, because it would be like “. . . giving up gold . . . or a million dollars” (Bernadette). Another woman, alluding to physician turnover, noted that she travelled to see a physician in another community rather than trying to find one locally because of the risk that local physician instability might lead to involuntary detachment: “. . . the problem is, they [family physicians] could only be here for a few months and then leave” (Wilda).

The majority of women who had transitioned spoke to the experience of detachment. While a patient or a physician can initiate detachment, the experiences described by participants in this phase of the transitioning trajectory were those resulting from physician-instigated separation. Accounts focused on two key features of detachment, both reflecting perceived failures of the system to adequately support patients during this process. The first feature was patient notification. Means of notification varied from calls, to letters, to no notice at all. There was a sense expressed by some participants that there was an unreasonable degree of randomness in how notifications were handled. One woman received no notification and learned of her doctor’s departure from a friend. Two women received letters outlining the upcoming departure of their physicians during previously scheduled appointments, raising questions about what might have happened if they had not had these appointments booked. As Anne explained: “…they didn’t send anything out. So if I had of been of good health, I would never have known.” Two other participants reported that their physicians ran newspaper ads announcing practice closure. One woman reflecting on her experience of being notified, described it as follows:

So that [reading about the practice closure in the paper] really kind of ticked me off, that that was how I found out. Mind you, he had a lot going on and I guess that was the way he felt he had to deal with it, was just put something in the paper and then if you happened to see it and you went in, then…that was fine (Kathy A).

The second feature of detachment described by participants was the relative absence of referrals to a new family doctor by the departing physicians. Only two women described being referred. One woman who had transitioned more than once was referred to an incoming physician in her earlier transition, but had not received a referral in her most recent experience. A second woman received a notice of practice closure suggesting that she try putting her name on the list of a new physician opening a practice. She did not do so, however, as she was then informally referred by her exiting physician who arranged for her to see his wife who was also a family physician. The remaining participants described detachments without a formal referral to a new family practice. One participant who had received a formal letter of notification pointed out that it “. . . didn’t make any suggestions of where to go for help or what to do” (Doreen).

The experiences of participants who had detached were echoed in the accounts of the four women who were anticipating transitioning because their long-term physicians were soon to retire. While one woman had been assured by her physician that he would help her find a new family doctor when the time came, the remaining women had discussed the upcoming detachment with their physicians and had not been presented with specific plans for how they should transition, nor been offered referrals. One participant ascribed the lack of referrals to the local physician shortage, noting that: “There was just nobody to refer to. They [the community] were already clamouring for doctors at that time” (Joye).

The absence of referrals, or other formal supports for transition, prompted some participants to describe their distress at detachment. One woman characterized her sense of being left to manage transition alone: “I felt like I was being abandoned” (Kim), while another expressed concern that detachment brought loss of the “peace and comfort of knowing that you have a doctor to call on” (Doreen). The absence of guidance, in tandem with awareness of the physician shortage, heightened anxiety. One woman, who had been through detachment before and was facing it again, summed it up this way:

So I don’t know…I’m at a loss of what I can do, because there are no doctors in [community] or any of the areas around [community]…the apprehension of not knowing whether you’re going to get one…gives you sleepless nights (Doreen).

Participants recognized that detachment, within the context of a physician shortage, could lead to prolonged periods without a physician. One woman, contemplating her doctor’s upcoming retirement, voiced this anxiety: “I’m scared to death, because there aren’t any. There aren’t any doctors. You can’t get one” (Leslie).

### Unattachment phase

Only three participants described transitions that did not include a phase of being unattached. One of these women had recently moved to a new community. She avoided this phase by maintaining her relationship with her original physician, a three hour drive away, for a period of year until her name rose to the top of a local physician’s wait list. This enabled her to move directly from detachment to attachment. The other two participants received referrals, allowing them to bypass unattachment.

In the absence of available local physicians and supports for reattachment, however, most detached participants spent considerable time without a regular family physician. Seven of the 10 women who had transitioned described phases of unattachment lasting several months to years. Three women were without a regular doctor for more than one year and two women were unattached for approximately four years. The inability to readily attach to a new family physician meant these participants had to assume responsibility for improvising interim means of receiving primary care. When describing her general sense of “winging it on your own” when managing care during this phase, one woman spoke to her sense of aloneness in navigating the health system: “. . . when . . . you’re ‘orphaned’, you know. . .you really do feel like an orphan” (Kathy A). Her reference to herself as an “orphan” reflects the emergence of this term in the common lexicon as a label for unattached patients – in particular for those who desire attachment but have been unable to find an available physician [38,39,75-77].

Three participants managed care during unattachment by relying on alternatives to a family physician, and found they were able to receive care that at least met the needs of their chronic illness. One woman, for example, sought care from her rheumatologist, a solution she felt worked well until she became ill with something beyond his scope of practice. She augmented this care by using a walk-in clinic. Two others elected to seek care with non-physician providers and both reported very positive experiences. A woman with diabetes who had been unattached for a year came to rely heavily on her diabetes educators – a nurse and a dietician. Her experience under the care of these educators was so positive that she continued to depend on them for diabetes care after she found a new family physician. Another participant sought care from a nurse practitioner and spoke highly of that experience, saying the nurse practitioner had given her “the best medical I ever had” (Yvonne).

Four women availed themselves of walk-in clinics during unattachment. Three relied exclusively on these clinics as their source of care during this phase, augmenting this strategy as necessary with visits to the Emergency Department at a local hospital. These women tended to speak less positively about their experiences than those who had developed other strategies for receiving primary care services, citing a range of concerns from inability to book ahead to restricted hours to long wait times:

The worst thing about that was you’d have to sit there for four or five hours. That was terrible . . . No, you cannot make an appointment . . .you walk in, you sit down, there could be 50 people sitting. Well, you’ve got to wait your turn (Sandy).

The women spoke of using these clinics reactively, out of necessity, to deal with specific, unavoidable health issues such as filling prescriptions. One woman described using clinics only for acute conditions like bladder infections and not for her chronic disease management, until her chronic symptoms became so acute she had no choice but to seek medical attention. Participants also spoke to the phenomenon of having serial physicians while using clinics – seeing whatever doctor happened to be available instead of having a consistent, continual care relationship with a single physician. All except one of the women who used walk-in clinics reported seeing multiple physicians, with one participant estimating that she saw four or five doctors over the period she used the clinic as her source of primary care.

Along with finding means to manage their health care needs, four of the participants who had experienced unattachment described unsuccessful search efforts during this phase, detailing on-going efforts to find a new regular physician who was accepting patients. One woman reported using a system resource – the Ontario College of Physicians and Surgeons website. She found the information on the site was outdated and that physicians she contacted who were listed as accepting patients, in fact were not. Another woman described a cycle of failed attempts: “I phoned a lot of places that people would suggest . . . and there was no way [to find a new doctor this way], every doctor is, ‘I’m not taking any more patients’” (Sandy). In an effort to exit unattachment two participants opted to expand the geographic domain of their search for a new physician and contemplated travelling for care beyond the confines of their own community: “I mean I looked up . . . anywhere that was get-at-able, to see if there were any doctors that were taking new patients” (Doreen).

### Attachment phase

Successfully identifying and engaging with a physician who was accepting patients was the principal activity described by participants who achieved attachment. Seven women described success in attachment, two of whom were the participants who had received referrals. For the remaining five, the element of chance that had surfaced in descriptions of detachment notification resurfaced in their accounts of finding a new physician. One woman reported hearing about a possible opening through the grapevine: “It was just luck that I heard that [doctor] was coming into town and I went right away and they gave me an appointment” (Anne). Another woman took a chance and approached a new physician in person, asking if she could be put on her waiting list. The physician instead accepted her immediately, directing her to call in and make an appointment. Yet another participant happened upon a newspaper advertisement run by a new doctor who was accepting patients, and went directly to the physician’s office and was taken on. The role of happenstance in attachment extended to the coincidental value of participants’ social networks to their efforts to identify an available physician. In one woman’s words, success in attaching often came down to having the right connections: “Anybody that gets in usually gets in either by family member or word of mouth” (Joye). The women also described a willingness to attach to any doctor that was available, pointing out that one could not be picky: “. . . if someone is willing to be your doctor, you accept them and put up with the things you don't like” (Kim).

For participants who succeeded in finding new physicians, the process of entering into a new relationship varied. Some reported completing applications and in-take forms, while others described having an initial, detailed interview appointment to go over their histories with the new physician. Participants also described learning the new rules of engagement, with varying success. One woman explained that her new physician expected patients to limit their appointments to talking about one or two key concerns and she had a hard time adjusting to this practice style. She noted that she did not "find the discussion part [of the appointment] happening any more" (Anne), adding that it was difficult with a chronic disease to only talk about one or two issues at a time. Many participants mentioned having to observe time limits to their appointments, ones not observed by their previous physicians. They described being allowed only 10 minutes per visit, leaving some with a sense that appointments were rushed: “You had to kind of cram… you felt that you had five or 10 minutes, and you'd better talk fast” (Kim).

### The transitioning trajectory

Most participants who had transitioned did not fully describe their involvement in each phase, focusing instead on experiences that were of particular significance to them. Two women did, however, provide a snapshot of their journeys through the full three-phased trajectory. The first learned of her physician’s departure during a previously scheduled appointment. She did not receive a referral and spent roughly a year as an unattached patient, availing herself of the services of a nurse practitioner in a neighbouring community. She eventually learned by word-of-mouth about a new local physician who was opening a practice, and called the office and was taken on. The second woman learned of her physician’s impending departure through a newspaper notice. She was also not provided with a referral and relied on her diabetes educators to assist her during her year of unattachment. She finally found a doctor taking patients through her social network: “I was just lucky to get into this doctor . . . and the only reason I did was my friend goes to that doctor’s daughter who is also a doctor. So she knew that he was coming in.” (Kathy A). These stories confirm a common theme of being largely left to figure out transitioning on one’s own.

## Discussion

The transitioning experiences completed or anticipated by participants revealed that losing a family physician marked the beginning of a transitioning trajectory involving at least two of three phases. Women who received referrals from their departing physicians, or had a lead on an available family physician experienced two phases, moving directly from detachment to attachment. Those who did not receive referrals typically went through a middle phase of unattachment. The descriptions of the first phase, detachment, centred on losing a family doctor due to practice closure with a focus on two key elements: how patients were notified about the closure and whether they were referred to, or given guidance about finding, a new family practice. When detachment included a referral, it led directly to the attachment phase, with participants being taken immediately into a new family practice. This, however, happened in a minority of cases. Most participants described an interim period of unattachment, during which they were without a regular physician and had to manage their primary care needs by employing a variety of tactics, most notably the use of alternative providers and walk-in clinics. About a third of the participants experienced a phase of unattachment lasting a year or longer, and these women described unsuccessful attempts to source a new family physician during this period. The attachment phase, which marks the endpoint of the transitioning trajectory, either flowed directly out of detachment as it did for the patients who were referred, or it followed unattachment. Either way, this phase was characterized by patients finding a successful means of identifying a physician with an open practice and being accepted into that practice.

It was evident from participants’ experiences that place of residence affected their ability to transition. Losing a family physician in communities contending with physician scarcity and turnover [11,12,65,66] appeared to elevate the transitioning process from a simple logistical task to one requiring endurance, ingenuity and compromise. As such, a straightforward transition between doctors proved the exception rather than the rule. The participants’ awareness that their communities suffered from a doctor shortage led some to make concessions in their approach to care, such as remaining with physicians they did not like, managing their chronic illnesses with alternative providers, or seeking and maintaining family physicians in other communities.

### Gaps in system support for transitioning patients

The participants tended to focus on challenges associated with transitioning and it was apparent that their need to compromise or improvise means of receiving care was engendered by perceived gaps in the health care system’s ability to support their transitions. Aside from the most critical and obvious gap in physician supply, the women identified other system lapses across the trajectory, and most participants described at least one experience during which they felt left to their own resources in managing their transition.

When discussing detachment, participants concentrated on system shortcomings regarding notification, referrals and guidance about transitioning. They spoke of passive notification tactics like newspaper advertisements, or in-office notices – leaving some women feeling that too much was left to chance. With so much at stake in losing a physician, these notification tactics were experienced as haphazard and posed a risk that patients might be deprived of valuable lead-time in their replacement search. Once notified, the pervasive lack of referrals may have been a function of the lack of available local physicians – a reality an exiting physician could not be expected to redress. The women, however, made no specific mention of other efforts made by their physicians to facilitate their transition, raising the question of where physician responsibility is understood to begin and end – by both patients and physicians.

During unattachment, most participants managed to find ways to receive interim care. Three women availed themselves of providers other than family physicians to manage their chronic illnesses. It was notable that these women were positive about the care they received, even while acknowledging the limitations of this tactic as a means of receiving the full range of primary care. The most widely employed tactic for using primary care services during unattachment, however, was the use of walk-in clinics and, as necessary, emergency departments. While these alternatives offered a means of system entry for care and enabled prescription refills and referrals to specialists, the participants focused on the downsides to walk-in clinics, in particular their wait times. For example, the walk-in experience dissuaded one patient from seeking routine care. She reported, despite her chronic illness, only seeking care at the clinic for acute conditions. And almost all the women spoke to the phenomenon of seeing multiple doctors when relying on walk-in clinics, which in effect denied them the opportunity for a continual care relationship – known to be associated with receipt of preventive care and positive health outcomes [21-25]. The activity of sourcing a new physician also began during unattachment, with this phase being characterized by unsuccessful efforts to identify an available physician. Search strategies varied, from randomly calling physician’s offices to canvassing neighbouring communities – but none were successful. The lone attempt at using a system resource, the College of Physicians and Surgeons website, also failed.

In describing the attachment phase, participants detailed successful efforts finding a new physician. Their accounts identified a need for self-reliance and good personal connections, with luck often playing a critical role in their successes. The means of ultimately achieving attachment were various, from chance encounters with physicians with capacity for new patients to leaning on family and friends. Across the transitioning trajectory the barriers to attachment encountered during detachment and unattachment, and the successful tactics employed in achieving attachment, all speak to an absence of generally available and well-publicized system resources to support patients seeking attachment.

### Implications for service delivery, primary care reform, and future research

The participants’ accounts of the challenges they encountered while transitioning shed light on health care system shortcomings. They also highlight opportunities for both short- and long-term system response. During detachment, the women identified issues with notification and lack of referrals. Guidelines issued by provincial or territorial Colleges of Physicians and Surgeons do specify that physicians ending a relationship should be “as helpful as possible” in assisting patients with finding new providers [78,79]. These guidelines, however, vary across jurisdictions, with some recommending assistance be provided to all patients [42] and others indicating this is necessary only for “selected patients” [41]. Some guidelines suggest that the provision of letters of introduction and lists of interim and emergency resources is adequate [43,44]. There is also variance in guidelines about notification: in some provinces or territories a newspaper notice is considered sufficient [42], while others advocate for letters to be mailed to patients’ homes [43,44]. The extent to which rural family physicians are aware of, or adherent to, guidelines is unclear. Research has identified multiple barriers to guideline uptake, from lack of awareness to cost [80]. Understanding how the system can best and most practicably support patients and physicians during detachment would be a useful, immediate point of focus for both health policymakers and provincial and territorial professional bodies.

The positive experiences receiving care from providers other than family physicians described by unattached participants lend support to the growing recognition that the traditional one-to-one patient-family physician relationship requires reconsideration beyond simply increasing numbers of family physicians. A critical thrust of primary care reform is the exploration of new delivery models [81,82], including the expansion of privileges and responsibilities for providers other than family physicians [83,84]. Participant experiences affirm reform initiatives such as nurse practitioner-led primary care clinics, which have opened in Ontario with considerable early success [76,83]. The less positive experiences of participants who used walk-in clinics and emergency departments warrant further exploration, as does the totality of the unattached patient experience [47,48]. While it has been postulated that unattached patients place an undue burden on emergency departments, the findings remain equivocal [85]. And not much is known about the long-term health consequences of relying on walk-in clinics and emergency departments for episodic primary care, especially for persons with chronic illness, although being unattached has been associated with a deficit in preventive care [16,17,47,48]. Given the large numbers of unattached Canadians, understanding how these stopgap service resources affect patient engagement with the system, as well as their on-going health management, is an important future research consideration.

During unattachment and attachment, participants’ efforts to identify available physicians underscore a need to evaluate the current system response to unattached patients. Responding to what is now widely recognized as a crisis in primary health care [8,9,86], Canadian provinces have starting to focus efforts on assisting patients seeking attachment [53-55]. The *Attachment Initiative* in British Columbia, for example, is piloting “locally appropriate strategies” in three communities, including clinics targeting unattached patients [87], while in Ontario the *Health Care Connect* registry links patients with available physicians. Evaluating the adequacy and effectiveness of such programs will take time; in the meantime it may prove worthwhile to explore options for creating a centrally maintained, well-publicized information portal that is both current and comprehensive, and includes information on available physicians and support programs for unattached patients, as well directories of local walk-in clinics.

### Limitations

There are three main limitations to this study. First, the interviews were conducted at a single point of time, although the phenomenon of transitioning is a longitudinal process. The retrospective nature of the interviews, in combination with the complexity of a process that unfolds over time, elevates the risk that key details may have been forgotten. This risk is of particular note for the few participants who described transitions that had taken place five to 10 years prior to the study. Second, although the original intent of the study was to examine the general experience of transitioning, the concept of a transitioning trajectory emerged from the data so each participant who had transitioned was not queried specifically about each phase. As a consequence, some participants’ narratives about the individual phases, and the trajectory in its entirety, are more complete than others. Third, the time constraints on the data collection period imposed limits on the sample size as participants had to be available for a face-to-face interview during a set period of time. While it was not possible to continue sampling until it was clear that saturation had been reached, the marked consistency in the participants’ descriptions, and their alignment with accounts of transitioning found in the popular media, affirmed the presence of both the phases and the trajectory.

## Conclusion

Many Canadians are unable to find an available family physician, and rural Canadians may have particular challenges doing so because physician supply in their communities tends to be limited and unstable [12,14]. Against this backdrop, this study explored chronically ill women’s perceptions and experiences of transitioning between doctors in a rural area with a known physician shortage [65,66]. The women characterized a distinct transitioning trajectory with three phases: detachment, unattachment and attachment. Their accounts revealed that moving through these phases was often difficult, largely because of a perceived lack support from the health care system.

Family physicians were recognized as a scarce resource. Awareness of the physician shortage created anxiety for those facing transition and caused three participants to avoid transitioning by remaining with physicians under less than ideal circumstances. The descriptions of those who had transitioned tended to validate this apprehension, confirming challenges across the trajectory that were largely associated with an absence of system resources to facilitate ready attachment to a new physician.

The participants’ accounts highlighted facets of the transitioning process that warrant research and policy attention. The concept of a transitioning trajectory deserves further investigation in order to develop a deeper understanding of the landscape of each phase, as well as the relationships and variations across the phases. Comparing trajectories that are initiated by a patient to those initiated by a physician, for example, may reveal different experiences both within and across phases. And in light of the national shortage of family physicians in Canada, using the trajectory to frame explorations of how both urban and rural patients, with various health needs, experience the phases of transitioning will afford an opportunity to explore additional possibilities for bolstering the system’s capacity to facilitate this process. The challenges that emerged from these women’s accounts signal a need for system attention to each phase of the trajectory, from notification through to identification of a new regular physician. Longer term, participants’ accounts of care while they were unattached point to a need to deepen our understanding of the role alternative sources of care play in primary care delivery. Considered collectively, these accounts of transitioning rendered a number of issues that merit consideration by rural policymakers and researchers, underscoring the importance of incorporating the patient experience into the on-going development and evaluation of primary care reform.

## Competing interests

The authors declare that they have no competing interests.

## Authors’ contributions

ER led the data analysis and the drafting of this article. VAC undertook the original data collection, participated in the data analysis and provided feedback on the article. LJG provided input on the analysis’ design and theorizing, and feedback on the article. All authors read and approved the final manuscript.

## Pre-publication history

The pre-publication history for this paper can be accessed here:

http://www.biomedcentral.com/1471-2296/13/125/prepub

## References

[B1] Health Analytics Branch - Health System Information Management and Investment Division Access to Primary Care in Ontario. 2009 2010 Toronto: Ontario Ministry of Health and Long-Term Care

[B2] The College of Family Physicians of Canada Family Practice. The Patient's Medical Home 2011 Mississauga: College of Family Physicians of Canada

[B3] The supply of physician services in OECD countries OECD Health Working Papers http://www.oecd.org/dataoecd/27/22/35987490.pdf

[B4] Sox HC Gabow P Feldbau G Scott CM Halvorson G The future of primary care Ann Intern Med 2003 138 230 12558363 10.7326/0003-4819-138-3-200302040-00016

[B5] Evans RG McGrail KM Richard III, Barer-Stoddart and the daughter of time Healthc Policy 2008 3 3 18 28 19305764 PMC2645150

[B6] Rosser WW The decline of family medicine as a career choice Can Med Assoc J 2002 166 11 1419 12054410 PMC111215

[B7] Canada S Access to a regular medical doctor, 2010 2011 Ottawa: Statistics Canada

[B8] Gulli C Lunau K Adding fuel to the doctor crisis Maclean's 2008 121 1 62 67

[B9] 4.1 million Canadians without family doctor: StatsCanhttp://www.cbc.ca/news/health/story/2008/06/18/doctor-statcan.html

[B10] Family Physician Shortage Estimates http://www.cma.ca/multimedia/CMA/Content_Images/Policy_Advocacy/Policy_Research/10-FP_Shortage-E.pdf

[B11] Rural Canada: access to health care http://publications.gc.ca/Collection-R/LoPBdP/BP/prb0245-e.htm

[B12] Pong RW Pitblado JR Geographic distribution of physicians in Canada: beyond how many and where 2005 Ottawa: Canadian Institute for Health Information

[B13] Physician Recruitment Strategy http://www.health.gov.sk.ca/adx/aspx/adxGetMedia.aspx?DocID=1633ff92-37fa-4ece-b798-1b8144d21420&MediaID=3712&Filename=physician-recruitment-2010.pdf&l=English

[B14] Tepper JD Schultz SE Rothwell DM Chan BTB Physician Services in Rural and Northern Ontario. ICES Investigative Report 2005 Toronto: Institute for Clinical Evaluative Sciences

[B15] Thommasen HV Physician retention and recruitment outside urban British Columbia B C Med J 2000 42 304 308

[B16] McIsaac WJ Fuller-Thomson E Talbot Y Does having regular care by a family physician improve preventive care? Can Fam Physician 2001 47 70 76 11212436 PMC2014713

[B17] Starfield B Is primary care essential? Lancet 1994 344 8930 1129 1133 10.1016/S0140-6736(94)90634-3 7934497

[B18] Haggerty JL Reid RJ Freeman GK Starfield BH Adair CE McKendry R Continuity of care: a multidisciplinary review BMJ 2003 327 7425 1219 1221 10.1136/bmj.327.7425.1219 14630762 PMC274066

[B19] Sparbel KJH Anderson MA Integrated literature review of continuity of care: part 1, conceptual issues J Nurs Scholarsh 2000 32 1 17 24 10.1111/j.1547-5069.2000.00017.x 10819734

[B20] Stokes T Tarrant C Mainous AG Schers H Freeman G Baker R Continuity of care: is the personal doctor still important? A survey of general practitioners and family physicians in England and Wales, the United States, and The Netherlands Ann Fam Med 2005 3 4 353 359 10.1370/afm.351 16046569 PMC1466891

[B21] Xu KT Usual source of care in preventive service use: a regular doctor versus a regular site Health Serv Res 2002 37 6 1509 1529 10.1111/1475-6773.10524 12546284 PMC1464041

[B22] Scobie A MacKinnon NJ Higgins S Etchegary H Church R The medical home in Canada: patient perceptions of quality and safety Healthc Manage Forum 2009 22 1 45 49 10.1016/S0840-4704(10)60292-X 19526887

[B23] Ryan S Riley A Kang M Starfield B The effects of regular source of care and health need on medical care use among rural adolescents Arch Pediatr Adolesc Med 2001 155 2 184 190 11177095 10.1001/archpedi.155.2.184

[B24] Bindman AB Grumbach K Osmond D Vranizan K Stewart AL Primary care and receipt of preventive services J Gen Intern Med 1996 11 5 269 276 10.1007/BF02598266 8725975

[B25] Blewett L Johnson P Lee B Scal P When a usual source of care and usual provider matter: adult prevention and screening services J Gen Intern Med 2008 23 9 1354 1360 10.1007/s11606-008-0659-0 18506542 PMC2518015

[B26] Starfield B Leiyu S The medical home, access to care, and insurance: a review of evidence Pediatrics 2004 113 1493 1498 15121917

[B27] Cloutier-Fisher D Skinner MW Levelling the playing field? Exploring the implications of managed competition for voluntary sector providers of long-term care in small town Ontario Health Place 2006 12 1 97 109 10.1016/j.healthplace.2004.10.012 16243684

[B28] Kenny A Duckett S A question of place: medical power in rural Australia Soc Sci Med 2004 58 6 1059 1073 10.1016/S0277-9536(03)00278-8 14723902

[B29] Pitblado JR Distribution and Internal Migration of Canada's Health Care Workforce 2007 Ottawa: Canadian Institute for Health Information

[B30] A portrait of seniors in Canadahttp://www.statcan.gc.ca/pub/89-519-x/89-519-x2006001-eng.pdf

[B31] Population Patterns of Chronic Health Conditions in Canada http://www.healthcouncilcanada.ca/docs/rpts/2007/outcomes2/Outcomes2PopulationPatternsFINAL.pdf

[B32] Sargious P Chronic Disease Prevention and Management Synthesis Series on Sharing Insights 2007 Ottawa: Health Canada

[B33] World Health Organization The Impact of Chronic Disease in Canada Facing the Facts 2005 Geneva: World Health Organization

[B34] World Health Organization Preventing chronic diseases: a vital investment 2005 Geneva: World Health Organization

[B35] Partnership for Solutions Chronic Conditions: Making the Case for Ongoing Care 2004 Baltimore: Johns Hopkins University, School of Hygiene and Public Health; The Robert Wood Johnson Foundation

[B36] Kellerman R Kirk L Principles of the patient-centered medical home Am Fam Physician 2007 76 6 774 775 17910291

[B37] The Robert Graham Center The Patient Centered Medical Home: History, Seven Core Features, Evidence and Transformational Change 2007 Washington, DC: The Robert Graham Center

[B38] More orphans soonhttp://www.thedailyobserver.ca/2009/07/03/more-orphans-soon

[B39] Centre makes difference in the lives of orphaned patientshttp://www.mykawartha.com/print/1052020

[B40] Dr. Rowan leaving Petawawa health centre for PRH http://www.thedailyobserver.ca/ArticleDisplay.aspx?e=1721478

[B41] Guideline: Closing a Practice http://www.cpsnb.org/english/Guidelines/ClosingaPractice.htm

[B42] Guideline - A Physician's Responsibilities When Permanently Closing a Medical Practice http://www.cpsnl.ca/default.asp?com=Policies&m=329&y=&id=12

[B43] Practice Management Considerations for Physicians Who Cease to Practice, Take an Extended Leave of Absence of Close Their Practice Due to Relocation http://www.cpso.on.ca/policies/policies/default.aspx?ID=1616

[B44] Leaving Practice https://http://www.cpsbc.ca/files/u6/Leaving-Practice.pdf

[B45] Misra-Hebert AD Kay R Stoller JK A review of physician turnover: rates, causes, and consequences Am J Med Qual 2004 19 2 56 66 10.1177/106286060401900203 15115276

[B46] Plomondon ME Magid DJ Steiner JF MaWhinney S Gifford BD Shih SC Grunwald GK Rumsfeld JS Primary care provider turnover and quality in managed care organizations Am J Manag Care 2007 13 8 465 472 17685827

[B47] Crooks V Agarwal G Harrison A Chronically ill Canadians' experience of being unattached to a family doctor: a qualitative study of patients in British Columbia BMC Fam Prac 2012 13 69 10.1186/1471-2296-13-69 PMC3412741 22799280

[B48] Hankivsky O Giesbrecht M Benoit C Crooks V Kaczorowski J Kazanjian A Identifying Canada's unattached patients: methodological challenges and research priorities 2012 Unpublished Manuscript

[B49] The Wait Starts Here. The Primary Care Wait Time Partnership http://cfpc.ca/uploadedFiles/Resources/Resource_Items/ENGLISH20PCWTP20FINAL20-20DECEMBER202009.pdf

[B50] Future of Family Medicine Project Leadership Committee The future of family medicine: a collaborative project of the family medicine community Ann Fam Med 2004 2 suppl 1 S3 S32 15080220 10.1370/afm.130 PMC1466763

[B51] Nutting PA Goodwin MA Flocke SA Zyzanski SJ Stange KC Continuity of primary care: to whom does it matter and when? Ann Fam Med 2003 1 3 149 155 10.1370/afm.63 15043376 PMC1466596

[B52] Pereira AG Pearson SD Patient attitudes toward continuity of care Arch Intern Med 2003 163 8 909 912 10.1001/archinte.163.8.909 12719199

[B53] A GP for Mehttp://www.gpscbc.ca/gp-me

[B54] Health Care Connect. Ontario's Unattached Patient Program http://www.chsrf.ca/Libraries/Picking_up_the_pace_files/joshua_tepper.sflb.ashx

[B55] Beaulieu M-D Rioux M Rocher G Samson L Boucher L Family practice: professional identity in transition. A case study of family medicine in Canada Soc Sci Med 2008 67 7 1153 1163 10.1016/j.socscimed.2008.06.019 18644668

[B56] Hutchison B Levesque J-F Strumpf E Coyle N Primary health care in Canada: systems in motion Milbank Q 2011 89 2 256 288 10.1111/j.1468-0009.2011.00628.x 21676023 PMC3142339

[B57] House of Commons Health Committee Patient and public involvement in the NHS. Third Report of Session 2006–7. Volume II 2007 London: Stationery Office

[B58] Schoen C Osborn R Huynh PT Doty M Davis K Zapert K Peugh J Primary care and health system performance: adults' experiences in five countries Health Aff (Millwood) 2004 Web Exclusives 23 W4-487 503 15513956 10.1377/hlthaff.w4.487

[B59] Patient-Centered Care An Introduction to What It Is and How to Achieve It. A Discussion Paper for the Saskatchewan Ministry of Health http://www.health.gov.sk.ca/Default.aspx?DN=49372758-a388-4e95-8fe2-043c18362458

[B60] Patient-Centered Care 2015: Scenarios, Vision, Goals, and Next Steps http://174.120.202.186/~pickerin/wp-content/uploads/2010/06/PCC-2015.pdf

[B61] Arthritis and Related Conditions in Ontario. Chapter 4. Primary and Specialist Care http://www.acreu.ca/pdf/ICES_atlas-ch4.pdf

[B62] van der Waal JM Bot SD Terwee CB van der Windt DA Schellevis FG Bouter LM Dekker J The incidences of and consultation rate for lower extremity complaints in general practice Ann Rheum Dis 2006 65 6 809 815 10.1136/ard.2005.036988 16269430 PMC1798168

[B63] 2010–2011 Year in Reviewhttp://www.arthritis.ca/local/files/pdf documents/About TAS/2011 English Annual Report Web_1.pdf

[B64] Weisman C Rich D Rogers J Crawford K Grayson C Henderson J Gender and patient satisfaction with primary care: tuning into women in quality measurement J Womens Health Gend Based Med 2000 9 6 657 666 10.1089/15246090050118189 10957754

[B65] Grey County putting out welcome mat for doctors http://www.theenterprisebulletin.com/ArticleDisplay.aspx?e=1251265&archive=true

[B66] Doctor shortage hard to pin down http://www.owensoundsuntimes.com/ArticleDisplay.aspx?e=3124141&archive=true

[B67] DiCicco-Bloom B Crabtree BF The qualitative research interview Med Educ 2006 40 4 314 321 10.1111/j.1365-2929.2006.02418.x 16573666

[B68] Creswell JW Research design: qualitative, quantitative, and mixed methods approaches 2009 Thousand Oaks, Calif: Sage Publications

[B69] Patton MQ How to use qualitative methods in evaluation 1987 Newbury Park, Calif: Sage Publications

[B70] Britten N Qualitative research: qualitative interviews in medical research BMJ 1995 311 6999 251 253 10.1136/bmj.311.6999.251 7627048 PMC2550292

[B71] Warren CAB Karner TX Discovering qualitative methods: field research interviews, and analysis 2005 Los Angeles, Calif: Roxbury

[B72] Boyatzis RE Transforming qualitative information: thematic analysis and code development 1998 Thousand Oaks, CA: Sage Publications

[B73] Morse JM Field P-A Qualitative research methods for health professionals 1995 Thousand Oaks: Sage Publications

[B74] Gladstone G The soul-destroying search for a family doctor The Globe and Mail 2011 L1

[B75] Follow up for "orphaned patients" http://www.cmpa-acpm.ca/cmpapd04/docs/resource_files/infosheets/2008/com_is0886-e.cfm

[B76] Nurse practitioner clinics aid orphan patientshttp://www.cbc.ca/news/canada/thunder-bay/story/2011/09/29/tby-doc-shortage.html

[B77] Orphan Patients: where's the solution? http://www.cbc.ca/whitecoat/episode/2009/12/02/orphan-patients-wheres-the-solution/

[B78] Guidelines for Ending the Physician-Patient Relationship http://www.cpsns.ns.ca/DesktopModules/Bring2mind/DMX/Download.aspx?Command=Core_Download&EntryId=14&PortalId=0&TabId=180

[B79] Ending the Physician-Patient Relationship http://www.cpso.on.ca/policies/policies/default.aspx?ID=1592

[B80] Disseminating Practice Guidelines to Physicians http://www.inspq.qc.ca/pdf/publications/048_Dissem_guidelines_ang.pdf

[B81] Green ME Hogg W Gray D Manuel D Roller M Maaten S Zhang Y Shortt SED Financial and work satisfaction: Impacts of participation in primary care reform on physicians in Ontario Healthc Policy 2009 5 2 e161 e176 21037819 PMC2805146

[B82] Primary health care teams and their impact on processes and outcomes of care http://epe.lac-bac.gc.ca/100/200/301/statcan/health_research_working_papers-e/02/82-622-XIE2008002.pdf

[B83] DiCenso A Bourgeault I Abelson J Martin-Misener R Kaasalainen S Carter N Harbman P Donald F Bryant-Lukosius D Kilpatrick K Utilization of nurse practitioners to increase patient access to primary healthcare in Canada – thinking outside the box Nurs Leadersh 2010 23 Special Issue 239 259 10.12927/cjnl.2010.22281 21478696

[B84] Eggertson L Naturopathic doctors gaining new powers Can Med Assoc J 2010 182 1 E29 E30 10.1503/cmaj.109-3112 19917661 PMC2802635

[B85] Myth: Emergency room overcrowding is caused by non-urgent cases http://www.chsrf.ca/PublicationsAndResources/Mythbusters/ArticleView/09-10-01/73a17f86-f6ea-4d82-b59e-d2305ff99a80.aspx 10.1258/jhsrp.2010.010310 20466755

[B86] Family doctors need more money, backup to improve patient care: studyhttp://www.cbc.ca/news/health/story/2007/10/11/doctors-study.html

[B87] Unattached Patientshttp://www.divisionsbc.ca/provincial/unattached

